# Genito-Urinary Surgery

**Published:** 1905-12-23

**Authors:** 


### GENITO-URINARY SURGERY.

(*Continued from page* 183.)

**The Röntgen Diagnosis of Ureteral Calculus.**—The diagnosis of ureteral calculi has been revolutionised by the application of the Röntgen method. C. L. Leonard [3] shows this in a recent paper on the subject. The method has demonstrated that ureteral calculi are much more common than was previously supposed. In a series of 330 cases examined, ureteral calculi were found more frequently than renal calculi in a proportion of forty cases to twenty-nine. Also the Röntgen method has led to a better differentiation of the symptoms respectively of ureteral and renal calculi, and with the additional accuracy in diagnosis the indications for and against operative treatment have been rendered clearer. In 26 of Leonard's cases the ureteral calculi were passed successfully without operation, and the *x*-rays may give material help in deciding whether the treatment should be expectant or operative. A calculus in the ureter is probably propelled by the hydrostatic pressure produced by the contraction of the distended kidney and ureter, not by the peristalsis of the ureter. There may be differences in the character of renal and of ureteral colic which suggest the presence of the calculus in the one or the other region. A dull ache in the lumbar region is common to both. The ureteral stone produces an increasing ache, due to the gradual distension of the kidney and ureter, until the acute attack of colic supervenes. The ureteral colic, in which the pain is more lancinating and more generally accompanied by nausea and vomiting than in renal colic, subsides gradually, unless the calculus is expelled. The prodromal stage and gradual subsidence of the lumbar pain differentiate ureteral colic from renal colic. During ureteral colic the pain radiates up to the kidney and downwards to the testicle, groin, scrotum, labium majus, urethra, inner side of the thigh, the knee, or even the foot. Also the psoas muscle is often irritated and flexed if the calculus is situated above the iliac artery. With calculus below that artery the symptoms are mainly vesical. By palpation a ureteral calculus can often be felt at the uretero-iliac junction, and this spot is then acutely tender. This pain and tenderness has to be differentiated from appendicitic and ovarian pain. The *x*-ray negative must be as reliable when it shows the absence of a calculus as when it shows the presence of such. Leonard holds that if an *x*-ray plate is taken with proper care and if the plate gives no indication of the presence of a calculus, then an operation for the detection of a calculus is unjustifiable. Reliable plates of this description can, of course, only be prepared by skiagraphists of experience. If the *x*-rays show that a stone is present in the kidney, this in itself is an indication for immediate operation. But if the calculus is in the ureter, then its size will show whether operative or expectant treatment is to be adopted, unless urgent complications are present. Where the calculus is small and there is a history of repeated attacks of colic, there is great probability that it will be passed by the natural channels. In such cases the attacks of colic should be welcomed as showing that progress is being made in the expulsion of the calculus.

**The Collection of Urine from each Kidney.**—This subject was recently discussed by J. W. T. Walker.[4] The instruments which may best be used for the purpose in question are the separators of Luys and of Cathelin and the catheterising cystoscopes of Nitze or Casper, or the "open method" of Kelly. The separators are designed to make a partition in the bladder dividing its cavity into two halves, into each of which one ureter opens and from each of which a separate catheter conducts the urine to external receptacles. With the catheterising cystoscopes fine catheters are passed, one into each ureter, by the aid of sight, the bladder being illuminated by the cystoscopic lamp. These instruments are required in two types of cases—namely, (1) When it is proposed to operate on one diseased kidney and information is desired with regard to the functional condition of the other kidney; and (2) when in the presence of renal hæmaturia or pyuria, it has to be ascertained which is the diseased side. It is more especially in cases of the first type that one or other of these instruments should be used. It is now a recognised canon in renal surgery that nephrectomy should not be performed unless it has previously been ascertained that there will be left behind another kidney which is at least fairly healthy. Many cases have been recorded in which a solitary kidney has been removed and many more still in which the kidney removed has been the one upon which the life of the patient depended. Such diseases as renal calculus, tubercular disease, and suppurative diseases of the kidney are frequently bilateral, and attempts must be made in the presence of such diseases to ascertain the functional capacity of that kidney which is surmised to be healthy or less diseased than the one upon which operation is proposed. In comparing the use of segregators with that of catheterising cystoscopes it may be pointed out that in using the former the results are apt to be fallacious unless a previous use of a cystoscope has demonstrated that the disease is renal or ureteral and not vesical; otherwise one-sided vesical disease might lead to the erroneous diagnosis of one-sided renal disease. Hence the catheterising cystoscope has the advantage that by its aid the disease may be first localised to the kidney and then the urine collected from each kidney. The danger of possible infection of the ureter or kidney by catheterisation seems very remote. Casper, in over 300 cases of ureteral catheterisation has not had a case of infection. The separators have the advantage of being more generally applicable and easier to use, and will, therefore, appeal to a wider field of workers.

[3] Lancet, June 17, 1905. [4] Pract., June 1905.

